# (2*E*)-3-(2-Bromo­phen­yl)-1-(5-bromo­thio­phen-2-yl)prop-2-en-1-one

**DOI:** 10.1107/S1600536812047939

**Published:** 2012-11-28

**Authors:** Suresh B. Vepuri, H. C. Devarajegowda, S. Jeyaseelan, S. Anbazhagan, Y. Rajendra Prasad

**Affiliations:** a Institute of Pharmacy, GITAM University, Visakhapatnam-45, Andhrapradesh, India; b Department of Physics, Yuvaraja’s College (Constituent College), University of Mysore, Mysore 570 005, Karnataka, India; c Karuna College of Pharmacy, Thirumittacode, Palakad 679 533, Kerala, India; d College of Pharmacy, Andhra University, Visakhapatnam, Andhrapradesh, India

## Abstract

The asymmetric unit of the title compound, C_13_H_8_Br_2_OS, contains two mol­ecules, in which the dihedral angles between the thio­phene and benzene rings are 10.5 (3) and 33.2 (4)°. There are no significant directional inter­actions in the crystal.

## Related literature

For further details of conformational modelling, see: Pascard (1995 ▶); Thomas *et al.* (2004 ▶). For related structures, see: Liang *et al.* (2011 ▶); Alex *et al.* (1993 ▶); Li & Su (1993 ▶).
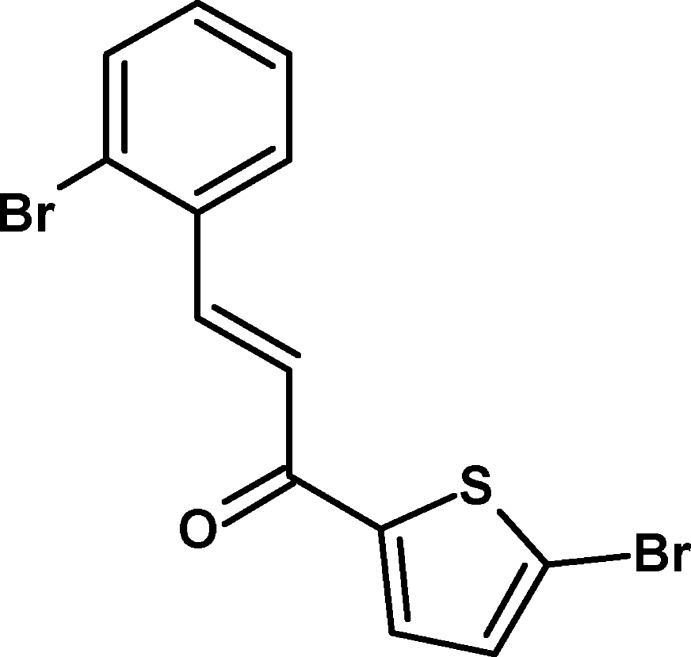


## Experimental

### 

#### Crystal data

C_13_H_8_Br_2_OS
*M* *_r_* = 372.07
Monoclinic, 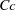

*a* = 34.524 (8) Å
*b* = 3.9994 (9) Å
*c* = 23.428 (5) Å
β = 126.804 (3)°
*V* = 2590.1 (10) Å^3^
*Z* = 8
Mo *K*α radiation
μ = 6.40 mm^−1^
*T* = 293 K
0.22 × 0.15 × 0.12 mm

#### Data collection

Oxford Diffraction Xcalibur diffractometer
Absorption correction: multi-scan (*CrysAlis PRO*; Oxford Diffraction, 2010 ▶) *T*_min_ = 0.228, *T*_max_ = 1.000
13574 measured reflections
5988 independent reflections
4266 reflections with *I* > 2σ(*I*)
*R*_int_ = 0.051

#### Refinement

*R*[*F* ^2^ > 2σ(*F* ^2^)] = 0.047
*wR*(*F* ^2^) = 0.109
*S* = 0.97
5988 reflections
308 parameters
2 restraints
H-atom parameters constrained
Δρ_max_ = 0.58 e Å^−3^
Δρ_min_ = −0.44 e Å^−3^
Absolute structure: Flack (1983 ▶), 2846 Friedel pairs
Flack parameter: 0.000 (13)

### 

Data collection: *CrysAlis PRO* (Oxford Diffraction, 2010 ▶); cell refinement: *CrysAlis PRO*; data reduction: *CrysAlis PRO*; program(s) used to solve structure: *SHELXS97* (Sheldrick, 2008 ▶); program(s) used to refine structure: *SHELXL97* (Sheldrick, 2008 ▶); molecular graphics: *ORTEP-3* (Farrugia, 2012 ▶); software used to prepare material for publication: *WinGX* (Farrugia, 2012 ▶).

## Supplementary Material

Crystal structure: contains datablock(s) I, global. DOI: 10.1107/S1600536812047939/hb6986sup1.cif

Structure factors: contains datablock(s) I. DOI: 10.1107/S1600536812047939/hb6986Isup2.hkl

Supplementary material file. DOI: 10.1107/S1600536812047939/hb6986Isup3.cml

Additional supplementary materials:  crystallographic information; 3D view; checkCIF report

## Figures and Tables

**Fig. 1.**
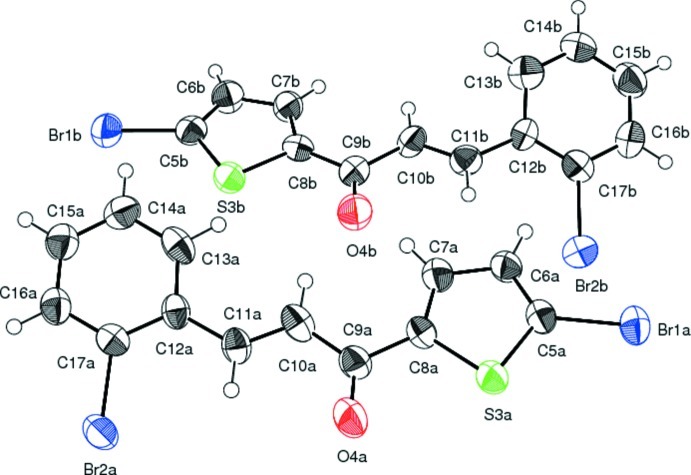
The molecular structure of the title compound. Displacement ellipsoids are drawn at the 50% probability level.

**Fig. 2.**
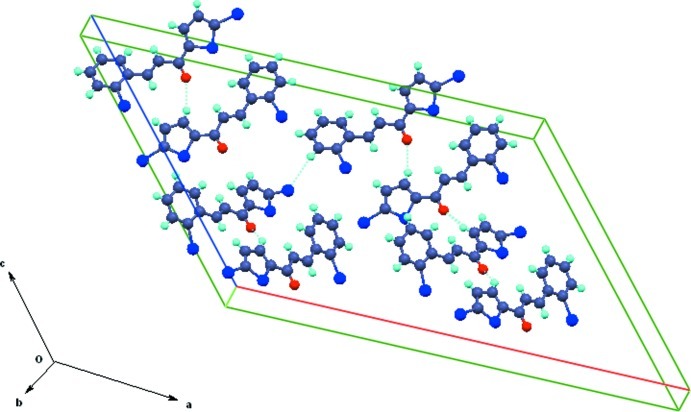
Packing of the molecules.

## supplementary crystallographic information

### Comment

Apart from numerous applications in basic research, crystal structure conformation of small molecules has always been the choice for binding energy calculations in molecular modeling during drug discovery process (Pascard, 1995). The reason is it provides coordinates for the most favorable stereo positions of the atoms in solid state.Use of the low energy conformation obtained in this bound state may provide good rationality in drug design study. As a part of our effort in designing lead compound for human aldose reductase inhibiton we are interested in studying the crystal structure conformation of (2*E*)-1-(5-bromothiophen-2-yl)-3-[4-(dimethylamino) phenyl]prop-2-en-1-one. In our docking studies the title chalcone has shown good binding affinity with dock score -9.490027 calculated by using GLIDE scoring (Thomas *et al.*, 2004) function from Schrodinger 9.2v molecular modeling suite.

The asymmetric unit of (2*E*)-1-(5-bromothiophen-2-yl)-3- (2-bromophenyl)prop-2-en-1-one, C_13_H_8_Br_2_OS, contain two molecules (Fig. 1). The five-membered thiophene rings (S3a\C5a\···C8a) & (S3b\C5b\···C8b) are not coplanar with the phenyl rings (C12*a*\C13*a*\···C17*a*) & (C12*b*\C13*b*\···C17*b*) system; the dihedral angle between the two planes are 10.5 (3)° and 33.2 (4)° of A and B molecules respectivelly. Bond distances and bond angles are in good agreement with those observed in related crystal structures (Liang *et al.*, 2011; Alex *et al.*, 1993; Li *et al.*, 1993). The packing of molecules in the crystal structure is depicted in Fig. 2.

### Experimental

A mixture of 2-acetyl-5-bromothiophene (0.01 mole) and 2-bromobenzaldehyde (0.01 mole) were stirred in ethanol (30 ml) and then an aqueous solution of potassium hydroxide (40%, 15 ml)was added to it. The mixture was kept over night at room temperature and then it was poured into crushed ice and acidified with dilute hydrochloric acid. The precipiteted chalcone was filtered and crystallized from ethanol as colourless prisms.

### Refinement

All H atoms were positioned at calculated positions C—H = 0.93 Å for aromatic H and refined using a riding model with *U*_iso_(H) = 1.2*U*_eq_(C)for aromatic H. Attempts to model non-merohedral twinning resulted in no improvement.

### Crystal data

**Table:**

C_13_H_8_Br_2_OS	*F*(000) = 1440
*M**_r_* = 372.07	*D*_x_ = 1.908 Mg m^−^^3^
Monoclinic, *C**c*	Melting point: 403 K
Hall symbol: C -2yc	Mo *K*α radiation, λ = 0.71073 Å
*a* = 34.524 (8) Å	Cell parameters from 5988 reflections
*b* = 3.9994 (9) Å	θ = 1.8–28.0°
*c* = 23.428 (5) Å	µ = 6.40 mm^−^^1^
β = 126.804 (3)°	*T* = 293 K
*V* = 2590.1 (10) Å^3^	Prism, colourless
*Z* = 8	0.22 × 0.15 × 0.12 mm

### Data collection

**Table:**

Oxford Diffraction Xcalibur diffractometer	5988 independent reflections
Radiation source: Mova (Mo) X-ray Source	4266 reflections with *I* > 2σ(*I*)
Mirror monochromator	*R*_int_ = 0.051
Detector resolution: 16.0839 pixels mm^-1^	θ_max_ = 28.0°, θ_min_ = 1.8°
ω scans	*h* = −44→44
Absorption correction: multi-scan (*CrysAlis PRO*; Oxford Diffraction, 2010)	*k* = −5→5
*T*_min_ = 0.228, *T*_max_ = 1.000	*l* = −30→30
13574 measured reflections	

### Refinement

**Table:**

Refinement on *F*^2^	Secondary atom site location: difference Fourier map
Least-squares matrix: full	Hydrogen site location: inferred from neighbouring sites
*R*[*F*^2^ > 2σ(*F*^2^)] = 0.047	H-atom parameters constrained
*wR*(*F*^2^) = 0.109	*w* = 1/[σ^2^(*F*_o_^2^) + (0.0425*P*)^2^] where *P* = (*F*_o_^2^ + 2*F*_c_^2^)/3
*S* = 0.97	(Δ/σ)_max_ < 0.001
5988 reflections	Δρ_max_ = 0.58 e Å^−^^3^
308 parameters	Δρ_min_ = −0.44 e Å^−^^3^
2 restraints	Absolute structure: Flack (1983), 2846 Friedel pairs
Primary atom site location: structure-invariant direct methods	Flack parameter: 0.000 (13)

### Special details

**Table:**

Geometry. All s.u.'s (except the s.u. in the dihedral angle between two l.s. planes) are estimated using the full covariance matrix. The cell s.u.'s are taken into account individually in the estimation of s.u.'s in distances, angles and torsion angles; correlations between s.u.'s in cell parameters are only used when they are defined by crystal symmetry. An approximate (isotropic) treatment of cell s.u.'s is used for estimating s.u.'s involving l.s. planes.
Refinement. Refinement of *F*^2^ against ALL reflections. The weighted *R*-factor *wR* and goodness of fit *S* are based on *F*^2^, conventional *R*-factors *R* are based on *F*, with *F* set to zero for negative *F*^2^. The threshold expression of *F*^2^ > 2σ(*F*^2^) is used only for calculating *R*-factors(gt) *etc*. and is not relevant to the choice of reflections for refinement. *R*-factors based on *F*^2^ are statistically about twice as large as those based on *F*, and *R*- factors based on ALL data will be even larger.

### Fractional atomic coordinates and isotropic or equivalent isotropic displacement parameters (Å^2^)

**Table:**

	*x*	*y*	*z*	*U*_iso_*/*U*_eq_	
Br1A	0.49316 (2)	0.58296 (18)	0.54216 (3)	0.05287 (19)	
Br2A	0.83387 (2)	−0.64813 (18)	0.74346 (3)	0.0554 (2)	
S3A	0.58670 (6)	0.2854 (4)	0.57198 (9)	0.0491 (4)	
O4A	0.6766 (2)	−0.0276 (16)	0.6126 (3)	0.0756 (17)	
C5A	0.5553 (2)	0.3956 (14)	0.6035 (4)	0.0416 (14)	
C6A	0.5789 (2)	0.3287 (16)	0.6733 (3)	0.0468 (16)	
H6A	0.5664	0.3709	0.6983	0.056*	
C7A	0.6247 (3)	0.1873 (16)	0.7037 (4)	0.0498 (17)	
H7A	0.6463	0.1295	0.7516	0.060*	
C8A	0.6338 (2)	0.1449 (14)	0.6553 (3)	0.0391 (14)	
C9A	0.6763 (3)	0.0017 (17)	0.6631 (4)	0.0466 (15)	
C10A	0.7161 (3)	−0.1089 (17)	0.7347 (4)	0.0531 (18)	
H10A	0.7143	−0.0709	0.7722	0.064*	
C11A	0.7544 (2)	−0.2607 (15)	0.7476 (4)	0.0445 (15)	
H11A	0.7537	−0.3056	0.7081	0.053*	
C12A	0.7978 (2)	−0.3670 (14)	0.8162 (4)	0.0404 (14)	
C13A	0.8033 (3)	−0.3032 (18)	0.8802 (4)	0.0549 (18)	
H13A	0.7785	−0.1964	0.8779	0.066*	
C14A	0.8448 (3)	−0.3956 (19)	0.9463 (4)	0.0544 (18)	
H14A	0.8472	−0.3529	0.9873	0.065*	
C15A	0.8820 (3)	−0.5488 (17)	0.9512 (4)	0.0535 (17)	
H15A	0.9097	−0.6088	0.9957	0.064*	
C16A	0.8787 (3)	−0.6158 (15)	0.8903 (4)	0.0495 (16)	
H16A	0.9043	−0.7155	0.8934	0.059*	
C17A	0.8364 (2)	−0.5300 (14)	0.8248 (3)	0.0405 (14)	
Br1B	0.84144 (3)	−1.0016 (2)	1.09708 (4)	0.0628 (2)	
Br2B	0.47871 (3)	−0.1788 (2)	0.68156 (4)	0.0673 (2)	
S3B	0.75065 (6)	−0.6749 (4)	0.96145 (9)	0.0487 (4)	
O4B	0.6618 (2)	−0.3491 (14)	0.8424 (3)	0.0657 (14)	
C5B	0.7785 (2)	−0.8334 (15)	1.0463 (3)	0.0461 (16)	
C6B	0.7510 (3)	−0.8115 (17)	1.0687 (4)	0.0541 (17)	
H6B	0.7601	−0.8887	1.1126	0.065*	
C7B	0.7066 (2)	−0.6571 (17)	1.0175 (3)	0.0492 (16)	
H7B	0.6832	−0.6189	1.0248	0.059*	
C8B	0.7003 (2)	−0.5683 (15)	0.9569 (3)	0.0418 (15)	
C9B	0.6608 (3)	−0.4037 (18)	0.8926 (4)	0.0468 (14)	
C10B	0.6193 (2)	−0.2910 (17)	0.8921 (3)	0.0478 (16)	
H10B	0.6217	−0.3147	0.9336	0.057*	
C11B	0.5802 (3)	−0.1621 (17)	0.8367 (4)	0.0484 (16)	
H11B	0.5789	−0.1439	0.7959	0.058*	
C12B	0.5375 (2)	−0.0406 (16)	0.8307 (3)	0.0421 (15)	
C13B	0.5440 (3)	0.0730 (18)	0.8926 (4)	0.0573 (18)	
H13B	0.5748	0.0715	0.9357	0.069*	
C14B	0.5053 (3)	0.187 (2)	0.8903 (4)	0.066 (2)	
H14B	0.5102	0.2568	0.9320	0.080*	
C15B	0.4599 (3)	0.198 (2)	0.8271 (5)	0.064 (2)	
H15B	0.4339	0.2748	0.8258	0.077*	
C16B	0.4530 (3)	0.0956 (18)	0.7660 (4)	0.0573 (19)	
H16B	0.4223	0.1065	0.7227	0.069*	
C17B	0.4913 (3)	−0.0237 (16)	0.7683 (4)	0.0484 (16)	

### Atomic displacement parameters (Å^2^)

**Table:**

	*U*^11^	*U*^22^	*U*^33^	*U*^12^	*U*^13^	*U*^23^
Br1A	0.0393 (4)	0.0547 (3)	0.0606 (5)	0.0062 (3)	0.0277 (4)	0.0033 (4)
Br2A	0.0560 (5)	0.0566 (4)	0.0650 (5)	0.0045 (3)	0.0424 (4)	−0.0061 (4)
S3A	0.0474 (10)	0.0591 (10)	0.0469 (9)	0.0091 (8)	0.0315 (9)	0.0035 (8)
O4A	0.060 (4)	0.114 (5)	0.066 (4)	0.028 (3)	0.045 (3)	0.009 (3)
C5A	0.035 (3)	0.034 (3)	0.055 (4)	−0.004 (3)	0.026 (3)	−0.005 (3)
C6A	0.047 (4)	0.057 (4)	0.043 (4)	0.009 (3)	0.031 (3)	0.004 (3)
C7A	0.049 (4)	0.052 (4)	0.048 (4)	0.010 (3)	0.029 (4)	0.004 (3)
C8A	0.042 (4)	0.034 (3)	0.048 (4)	0.003 (3)	0.030 (3)	0.002 (3)
C9A	0.046 (4)	0.052 (4)	0.051 (4)	0.002 (3)	0.034 (4)	0.000 (3)
C10A	0.054 (5)	0.053 (4)	0.069 (5)	0.006 (3)	0.045 (4)	0.003 (4)
C11A	0.037 (4)	0.049 (3)	0.054 (4)	0.001 (3)	0.030 (3)	0.004 (3)
C12A	0.035 (3)	0.037 (3)	0.051 (4)	0.000 (3)	0.027 (3)	0.004 (3)
C13A	0.054 (5)	0.058 (4)	0.074 (5)	0.004 (4)	0.050 (4)	0.000 (4)
C14A	0.053 (5)	0.062 (4)	0.045 (4)	−0.007 (4)	0.028 (4)	−0.003 (3)
C15A	0.046 (4)	0.055 (4)	0.056 (4)	−0.001 (3)	0.029 (4)	0.000 (3)
C16A	0.040 (4)	0.044 (4)	0.061 (4)	0.000 (3)	0.028 (4)	−0.001 (3)
C17A	0.048 (4)	0.031 (3)	0.050 (4)	−0.002 (3)	0.033 (4)	0.000 (3)
Br1B	0.0518 (5)	0.0594 (4)	0.0590 (5)	0.0080 (4)	0.0235 (4)	−0.0050 (4)
Br2B	0.0649 (6)	0.0759 (5)	0.0509 (4)	0.0063 (4)	0.0293 (4)	−0.0050 (4)
S3B	0.0485 (10)	0.0561 (10)	0.0487 (9)	−0.0001 (8)	0.0330 (9)	0.0004 (8)
O4B	0.064 (4)	0.090 (4)	0.050 (3)	0.016 (3)	0.037 (3)	0.014 (3)
C5B	0.049 (4)	0.034 (3)	0.048 (4)	−0.005 (3)	0.025 (4)	−0.004 (3)
C6B	0.057 (5)	0.058 (4)	0.049 (4)	0.004 (4)	0.033 (4)	0.008 (3)
C7B	0.042 (4)	0.064 (4)	0.048 (4)	−0.003 (3)	0.030 (4)	−0.002 (3)
C8B	0.050 (4)	0.039 (3)	0.043 (4)	−0.003 (3)	0.032 (3)	−0.003 (3)
C9B	0.045 (4)	0.049 (3)	0.042 (3)	−0.001 (3)	0.024 (3)	0.000 (3)
C10B	0.040 (4)	0.060 (4)	0.037 (3)	0.000 (3)	0.019 (3)	0.005 (3)
C11B	0.050 (4)	0.056 (4)	0.045 (4)	−0.002 (3)	0.032 (4)	0.000 (3)
C12B	0.035 (4)	0.044 (4)	0.040 (3)	−0.001 (3)	0.019 (3)	0.006 (3)
C13B	0.058 (5)	0.061 (4)	0.062 (5)	−0.010 (4)	0.041 (4)	−0.003 (4)
C14B	0.072 (6)	0.077 (6)	0.056 (5)	0.008 (5)	0.042 (5)	−0.009 (4)
C15B	0.059 (5)	0.068 (5)	0.076 (6)	0.003 (4)	0.046 (5)	−0.004 (4)
C16B	0.046 (5)	0.061 (5)	0.056 (4)	0.008 (3)	0.026 (4)	0.011 (4)
C17B	0.059 (5)	0.043 (4)	0.055 (4)	0.005 (3)	0.040 (4)	0.007 (3)

### Geometric parameters (Å, º)

**Table:**

Br1A—C5A	1.880 (6)	Br1B—C5B	1.870 (7)
Br2A—C17A	1.913 (6)	Br2B—C17B	1.909 (7)
S3A—C5A	1.694 (6)	S3B—C5B	1.729 (7)
S3A—C8A	1.723 (7)	S3B—C8B	1.731 (7)
O4A—C9A	1.197 (8)	O4B—C9B	1.217 (9)
C5A—C6A	1.346 (9)	C5B—C6B	1.333 (10)
C6A—C7A	1.407 (9)	C6B—C7B	1.403 (10)
C6A—H6A	0.9300	C6B—H6B	0.9300
C7A—C8A	1.354 (9)	C7B—C8B	1.350 (9)
C7A—H7A	0.9300	C7B—H7B	0.9300
C8A—C9A	1.481 (9)	C8B—C9B	1.449 (10)
C9A—C10A	1.461 (10)	C9B—C10B	1.496 (11)
C10A—C11A	1.316 (9)	C10B—C11B	1.293 (9)
C10A—H10A	0.9300	C10B—H10B	0.9300
C11A—C12A	1.458 (9)	C11B—C12B	1.478 (10)
C11A—H11A	0.9300	C11B—H11B	0.9300
C12A—C17A	1.385 (9)	C12B—C17B	1.376 (9)
C12A—C13A	1.416 (10)	C12B—C13B	1.402 (10)
C13A—C14A	1.388 (11)	C13B—C14B	1.382 (11)
C13A—H13A	0.9300	C13B—H13B	0.9300
C14A—C15A	1.364 (10)	C14B—C15B	1.368 (11)
C14A—H14A	0.9300	C14B—H14B	0.9300
C15A—C16A	1.388 (10)	C15B—C16B	1.367 (10)
C15A—H15A	0.9300	C15B—H15B	0.9300
C16A—C17A	1.387 (9)	C16B—C17B	1.376 (10)
C16A—H16A	0.9300	C16B—H16B	0.9300
			
C5A—S3A—C8A	90.4 (3)	C5B—S3B—C8B	90.4 (3)
C6A—C5A—S3A	113.4 (5)	C6B—C5B—S3B	113.1 (5)
C6A—C5A—Br1A	126.2 (5)	C6B—C5B—Br1B	127.1 (5)
S3A—C5A—Br1A	120.4 (4)	S3B—C5B—Br1B	119.8 (4)
C5A—C6A—C7A	112.0 (6)	C5B—C6B—C7B	111.4 (6)
C5A—C6A—H6A	124.0	C5B—C6B—H6B	124.3
C7A—C6A—H6A	124.0	C7B—C6B—H6B	124.3
C8A—C7A—C6A	112.2 (6)	C8B—C7B—C6B	114.8 (6)
C8A—C7A—H7A	123.9	C8B—C7B—H7B	122.6
C6A—C7A—H7A	123.9	C6B—C7B—H7B	122.6
C7A—C8A—C9A	130.8 (6)	C7B—C8B—C9B	132.2 (7)
C7A—C8A—S3A	112.0 (5)	C7B—C8B—S3B	110.4 (5)
C9A—C8A—S3A	117.3 (5)	C9B—C8B—S3B	117.4 (5)
O4A—C9A—C10A	123.1 (7)	O4B—C9B—C8B	122.2 (7)
O4A—C9A—C8A	120.8 (7)	O4B—C9B—C10B	121.4 (7)
C10A—C9A—C8A	116.1 (6)	C8B—C9B—C10B	116.4 (6)
C11A—C10A—C9A	121.9 (7)	C11B—C10B—C9B	123.1 (6)
C11A—C10A—H10A	119.0	C11B—C10B—H10B	118.5
C9A—C10A—H10A	119.0	C9B—C10B—H10B	118.5
C10A—C11A—C12A	128.0 (7)	C10B—C11B—C12B	127.4 (6)
C10A—C11A—H11A	116.0	C10B—C11B—H11B	116.3
C12A—C11A—H11A	116.0	C12B—C11B—H11B	116.3
C17A—C12A—C13A	115.1 (6)	C17B—C12B—C13B	116.6 (6)
C17A—C12A—C11A	124.1 (6)	C17B—C12B—C11B	124.9 (6)
C13A—C12A—C11A	120.8 (6)	C13B—C12B—C11B	118.4 (6)
C14A—C13A—C12A	121.8 (7)	C14B—C13B—C12B	120.9 (7)
C14A—C13A—H13A	119.1	C14B—C13B—H13B	119.5
C12A—C13A—H13A	119.1	C12B—C13B—H13B	119.5
C15A—C14A—C13A	120.3 (7)	C15B—C14B—C13B	120.6 (7)
C15A—C14A—H14A	119.9	C15B—C14B—H14B	119.7
C13A—C14A—H14A	119.9	C13B—C14B—H14B	119.7
C14A—C15A—C16A	120.5 (7)	C16B—C15B—C14B	119.4 (7)
C14A—C15A—H15A	119.8	C16B—C15B—H15B	120.3
C16A—C15A—H15A	119.8	C14B—C15B—H15B	120.3
C17A—C16A—C15A	118.2 (7)	C15B—C16B—C17B	120.2 (7)
C17A—C16A—H16A	120.9	C15B—C16B—H16B	119.9
C15A—C16A—H16A	120.9	C17B—C16B—H16B	119.9
C12A—C17A—C16A	124.1 (6)	C12B—C17B—C16B	122.3 (6)
C12A—C17A—Br2A	120.4 (5)	C12B—C17B—Br2B	119.7 (5)
C16A—C17A—Br2A	115.5 (5)	C16B—C17B—Br2B	118.0 (6)
			
C8A—S3A—C5A—C6A	−0.3 (5)	C8B—S3B—C5B—C6B	1.0 (5)
C8A—S3A—C5A—Br1A	−179.1 (4)	C8B—S3B—C5B—Br1B	−177.5 (4)
S3A—C5A—C6A—C7A	1.0 (8)	S3B—C5B—C6B—C7B	−1.3 (8)
Br1A—C5A—C6A—C7A	179.7 (5)	Br1B—C5B—C6B—C7B	177.1 (5)
C5A—C6A—C7A—C8A	−1.4 (9)	C5B—C6B—C7B—C8B	1.0 (9)
C6A—C7A—C8A—C9A	−178.8 (6)	C6B—C7B—C8B—C9B	−179.3 (7)
C6A—C7A—C8A—S3A	1.2 (8)	C6B—C7B—C8B—S3B	−0.2 (8)
C5A—S3A—C8A—C7A	−0.5 (5)	C5B—S3B—C8B—C7B	−0.4 (5)
C5A—S3A—C8A—C9A	179.4 (5)	C5B—S3B—C8B—C9B	178.8 (5)
C7A—C8A—C9A—O4A	176.7 (8)	C7B—C8B—C9B—O4B	−178.9 (8)
S3A—C8A—C9A—O4A	−3.2 (9)	S3B—C8B—C9B—O4B	2.1 (10)
C7A—C8A—C9A—C10A	−1.3 (11)	C7B—C8B—C9B—C10B	3.5 (11)
S3A—C8A—C9A—C10A	178.7 (5)	S3B—C8B—C9B—C10B	−175.5 (5)
O4A—C9A—C10A—C11A	−2.6 (11)	O4B—C9B—C10B—C11B	7.4 (12)
C8A—C9A—C10A—C11A	175.4 (6)	C8B—C9B—C10B—C11B	−175.0 (7)
C9A—C10A—C11A—C12A	176.2 (6)	C9B—C10B—C11B—C12B	−179.4 (7)
C10A—C11A—C12A—C17A	179.1 (7)	C10B—C11B—C12B—C17B	−154.3 (7)
C10A—C11A—C12A—C13A	−1.7 (10)	C10B—C11B—C12B—C13B	27.0 (11)
C17A—C12A—C13A—C14A	0.6 (10)	C17B—C12B—C13B—C14B	1.6 (10)
C11A—C12A—C13A—C14A	−178.6 (6)	C11B—C12B—C13B—C14B	−179.5 (7)
C12A—C13A—C14A—C15A	0.8 (11)	C12B—C13B—C14B—C15B	−1.4 (12)
C13A—C14A—C15A—C16A	−0.4 (11)	C13B—C14B—C15B—C16B	−0.1 (13)
C14A—C15A—C16A—C17A	−1.5 (10)	C14B—C15B—C16B—C17B	1.3 (12)
C13A—C12A—C17A—C16A	−2.6 (9)	C13B—C12B—C17B—C16B	−0.5 (10)
C11A—C12A—C17A—C16A	176.5 (6)	C11B—C12B—C17B—C16B	−179.2 (7)
C13A—C12A—C17A—Br2A	178.0 (5)	C13B—C12B—C17B—Br2B	−179.1 (5)
C11A—C12A—C17A—Br2A	−2.9 (8)	C11B—C12B—C17B—Br2B	2.2 (9)
C15A—C16A—C17A—C12A	3.1 (9)	C15B—C16B—C17B—C12B	−1.0 (11)
C15A—C16A—C17A—Br2A	−177.4 (5)	C15B—C16B—C17B—Br2B	177.6 (6)
